# Zoonotic Gastrointestinal Parasites of Baboons (*Papio anubis*) in the Shai Hill Reserve in Ghana

**DOI:** 10.1155/2020/1083251

**Published:** 2020-03-11

**Authors:** John Asiedu Larbi, Stephen Akyeampong, Amina Abubakari, Seth Offei Addo, Dinah Okoto, Henry Hanson

**Affiliations:** ^1^Department of Theoretical and Applied Biology, College of Science, KNUST, PMB KNUST, Kumasi, Ghana; ^2^Department of Microbiology, Kumasi Technical University, P.O. Box 854, Kumasi, Ghana; ^3^Parasitology Department, Noguchi Memorial Institute for Medical Research, University of Ghana, P.O. Box LG 581, Legon, Accra, Ghana; ^4^Kumasi Centre for Collaborative Research into Tropical Medicine, UPO, PMB KNUST, Kumasi, Ghana

## Abstract

Several intestinal parasites of baboons are of zoonotic importance, especially where there is considerable interaction between the baboons and humans. The identification of gastrointestinal parasite infections of baboons (*Papio anubis*) was undertaken at the Shai Hills Reserve Resort, Ghana. A total of 51 faecal samples were collected randomly from individual baboons (51) and examined for cysts, eggs, and larvae of parasites of medical importance, using the direct saline smear and formol-ether concentration technique. The baboons were in 7 troops and were grouped into juveniles, subadults, and adults. The survey showed that 92% of the baboon samples examined were infected with at least one or more parasite(s) of medical importance. Hookworm had the highest prevalence of 38.39% followed by *Ascaris lumbricoides* (22.32%), whilst *Diphyllobotrium latum* recorded the lowest prevalence of 0.89%. Adults had the highest prevalence of *A. lumbricoides* (35.42%). Hookworm (47.92%) occurred most in the juveniles. This is the first report of intestinal parasitic infection in baboons within the Shai Hills Reserve. The results showed a high level of multiparasitism in the nonhuman primates, thus the need for possible interventions to maintain ecosystem health and control the infections as they may be a potential source of transmission to humans.

## 1. Introduction

Intestinal parasites are among the most important parasites of humans as well as other nonhuman primates, especially the main soil-transmitted helminths: *A. lumbricoides*, *Trichuris trichiura*, *Strongyloides stercoralis*, and hookworm [1]. The adult worms of these parasites inhabit the intestine of their host, producing eggs which are usually discharged in faeces of the host [2]. The eggs/cysts or larvae can be transferred to other animals or humans that may directly or indirectly come into contact with the infected faeces of the host organism [3], usually by ingestion or penetration of infective stages.

In sub-Saharan Africa, where the climate is hot coupled with generally poor hygienic conditions and in most cases poor sewage disposal, intestinal parasitism is common. Infection with these parasites results in morbidity and severe infection, especially in children. It generally causes diminished immunity and leaves their patients susceptible to many other diseases. Nonhuman primates also harbour a variety of gastrointestinal parasites which can cause blood loss, tissue damage, spontaneous abortion, congenital malformations, and death [4].

The baboon is the largest nonhominid member of the primate order belonging to the family Cercopithecidae (Old World monkeys). Olive baboons are group-living, omnivorous, habitat generalists with the largest geographic distribution of all African primates [5]. There are five species of baboons found in 25 equatorial countries of Africa, extending from Mali, eastwards to Ethiopia and Tanzania, with isolated populations found in some mountainous regions of the Sahara, mostly inhabiting savannahs, steppes, and forests (http://www.wikipedia.com/olivebaboons). Baboons have long dog-like muzzles, close-set eyes, and heavy powerful jaws and are known to raid human dwellings as well as prey on sheep and goats [6]. Baboons as nonhuman primates are also most affected by the same or different species of the common intestinal parasites of humans. These infections, although not obvious as viral infections, threaten the existence of the baboons and could as well be indicators of population health. The interaction of these baboons with humans also create avenues for zoonotic and anthropozoonotic transmission of gastrointestinal parasites. The similarities in physiology, behaviour, and genetics between humans and baboons increase the susceptibility to cross-species infections by the same parasites and could pose a serious threat to both humans and baboons [7].

The Shai Hills Reserve, like many reserves and parks, are areas where there is usually the day to day contact between tourists, workers, indigenous people, and the various animals that serve as an attraction. It serves as both a conservational site and a tourist attraction with interactions between some animals in the reserve and people living in and around the reserve as well as tourists. The people of Shai go to the mountains (inhabited by the baboons) believed to be their ancestral home to pay homage to their ancestors every year and interact with the baboons as “pets.” With only slight physiological differences between humans and baboons coupled with the behaviour of the baboons, there is the possibility of zoonotic transmission of infections, more so in the case where helminth infections are more common in baboon populations living in savannah areas [8]. Since some of these helminths, such as *A. lumbricoides*, hookworm, and *T. trichiura* are soil-transmitted, the human population is prone to these infections from the baboons and vice versa as they interact. Dry conditions tend to select baboons as an ideal harbour for helminths which complete their life cycles without prolonged exposure to abiotic environmental conditions [9]. Data on parasite types and prevalence in the baboons within the reserve is, however, rare. It is, therefore, necessary to generate baseline data on parasite infections in order to effectively maintain ecosystem health and also manage the health of the baboons which are the main source of attraction at the reserve and, based on this information, be able to adopt measures to examine and prevent any possibility of cross infection.

## 2. Materials and Methods

### 2.1. Study Area

**Figure 1:**
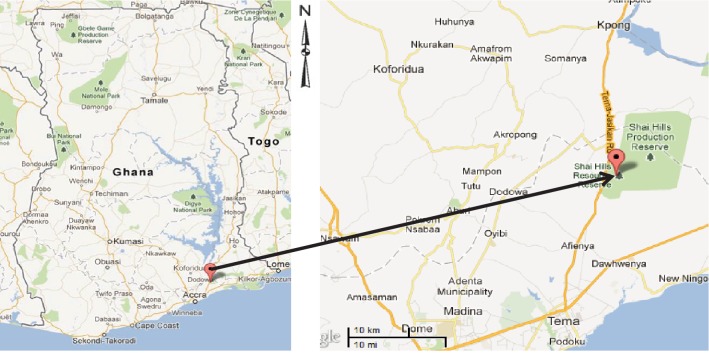
Map of Ghana showing the location of the Shai Hill Reserve (source: https://www.googlemaps.com).

Shai Hills Reserve is a 51-square kilometre wildlife park on latitude 5°53′30″N and longitude 0°3′38″E (http://www.wikimapia.org/21051944/Shai-hills-monkey-sanctuary) located at Doryumu in the Dangme West District of the Greater Accra Region, Ghana (Figure 1). It is a rocky mountainous area with a grassland plain around it. The vegetation is a coastal savannah type with high grass cover and scattered trees over the stretch of land. Generally, the hill slopes have savannah woodland and the hillsides in the central valley are characterised by thickets and dry forest [10]. The land is clayey (dark) with interspersed gravels at certain places, and the climate is usually characterised by moderate temperatures, fairly high humidity, and also low rainfall covering two rainy seasons [10]. It is the home of the olive baboons, antelopes, snakes, different species of birds, and other animals. There is a dam within the reserve from which the animals drink. Shai Hills Reserve is also the ancestral home of the Shai people, where the local people celebrate the “Ngmayem” festival that brings the community to the park's caves for the performance of ethnic rituals.

### 2.2. Study Design

The cross-sectional study was conducted by randomly selecting seven troops from 3 main territories of baboons, based on where they played and searched for food. Three troops (troops 1, 2, and 3) were selected randomly from those that sleep around the working area of the reserve, two (troops 4 and 5) from those that sleep at the mountain base, and the remaining troops (troops 6 and 7) were from the mountain top.

### 2.3. Sample Collection and Analysis

Faecal samples of the baboons were collected from the Shai Hills Reserve from February to March 2009. The baboons were trailed and fresh stool samples were collected using sterile spatulas into sample bottles. Each faecal sample taken was representative of a baboon. The samples were then preserved in isotonic formal saline and transported to the laboratory for examination within 12 hours. The baboons (samples) were grouped into juveniles (the very small ones), subadults (those that had not reached the adult stage and are not juveniles), and the adults. The saline wet mount method (thin saline smear) and the formol-ether concentration technique [11] were used for sample preparation, and the eggs, cysts, and larvae were identified microscopically with help from trained personnel using WHO [12] bench aids in the laboratory. The concentrated technique (formol-ether concentration technique) was done since the thin saline smear may not detect parasites in low egg burden samples. Briefly, 2 g of each stool sample was emulsified first in 3 ml of 10% formal saline solution and then another 4 ml of 10% formal saline was added and stirred. The resulting suspension was then sieved through a wet gauze into a 15 ml test tube, topped up to the 7 ml mark where necessary, 3 ml of diethyl-ether added to the suspension, mixed properly for about a minute, and centrifuged for 5 minutes at 2000 rpm. The supernatant was decanted, a drop of sediment placed on a clean dry microscope slide, a drop of Lugol's iodine added to enhance visibility, and covered with a coverslip. Each slide was examined systematically with a 10x objective lens starting at one corner of the smear and then with a 40x high-power objective lens. The samples were prepared and examined in duplicates.

## 3. Results

### 3.1. The General Prevalence of Infection

**Figure 2:**
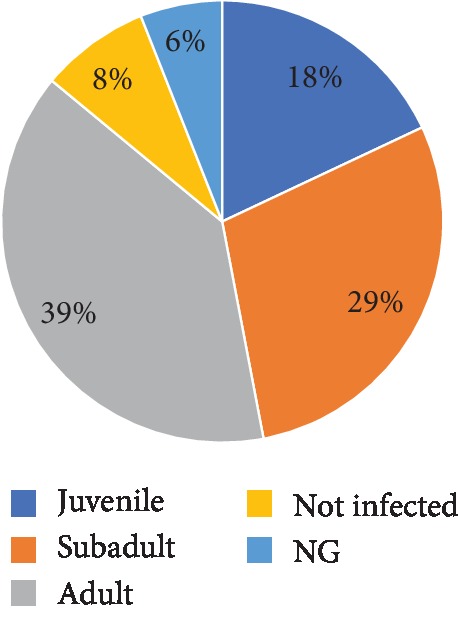
Overall prevalence of intestinal parasites in the various groups of baboons.

The total prevalence of infection was 92%. The parasites recorded consisted of both protozoans and helminths. These include *A. lumbricoides*, hookworm, *T. trichiura*, *Strongyloides stercoralis*, *Entamoeba histolytica*/*dispar*, *D. latum*, *Iodamoeba buetschlii*, and *Entamoeba coli.*Figure 2 displays the parasite prevalence in the different baboon age groups. The adults were observed to have the highest prevalence of intestinal parasites (39%), while the juveniles (18%) had the least. Infections (6%) were detected in samples collected from baboons which could not be placed in any of the groups (NG) (Figure 2).

### 3.2. Prevalence of the Various Intestinal Parasites

**Figure 3:**
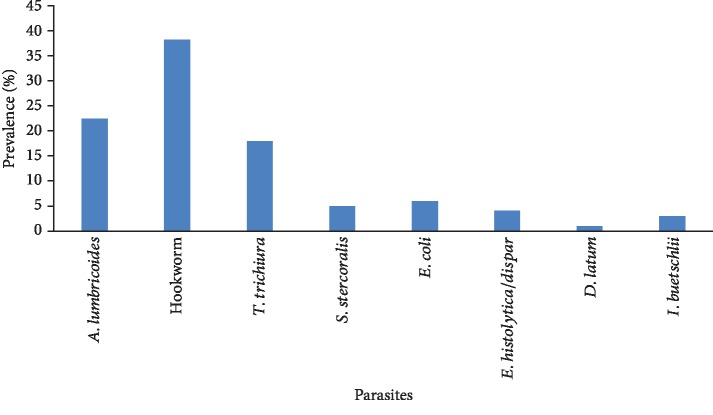
Prevalence of various intestinal parasites identified.

Hookworm infections were of the highest prevalence (38.4%) in the baboons, while *D. latum* had the least prevalence (0.9%) (Figure 3). The most prevalent parasite in both adult (35.4%) and subadult (10.4%) baboons was *A. lumbricoides*, while hookworm was the most prevalent parasite in the juveniles (47.9%). Multiple parasitic infections were observed in 11 (21.6%) of the sampled baboons with different parasites including *A. lumbricoides*, hookworm, *T. trichiura*, *Entamoeba histolytica*/*dispar*, *Iodamoeba buetschlii*, and *Entamoeba coli.*Table 1 summarizes the distribution of the various intestinal parasites by age groups for the baboons.

### 3.3. Distribution of the Various Parasites in the Different Groups

The highest prevalence for *A. lumbricoides* was recorded in adults (35.4%). The subadults recorded 10.4% for *A. lumbricoides*, while the juveniles recorded 8.3% (Table 1). For hookworm, the juveniles recorded the highest infection of 47.9%, followed by the adults with 20.9%, with the subadults recording the least (12.5%).

## 4. Discussion

Gastrointestinal parasites belonging to eight taxa (four nematodes and four protozoa) were identified in the baboon population studied. These consisted of *A. lumbricoides*, hookworm, *T. trichiura*, *Strongyloides stercoralis*, *E. histolytica*/*dispar*, *D. latum*, *Iodamoeba buetschlii*, and *Entamoeba coli*, most of which are pathogenic and could cause considerable damage to the host, especially during heavy infections. The findings of this study were similar to other studies which also found among others *Schistosoma mansoni*, *Trichostrongylus* sp., *Balantidium coli*, and *Blastocystis hominis* in addition [13, 14].

The high prevalence (92%) of intestinal parasites in baboons at Shai Hills can be compared to a similar study in olive baboons at the Mole National Park which recorded a prevalence rate of 93% [15]. The observed high prevalence in the present study could be attributed to the foraging behaviour and high mobility of the baboons which predisposes them to frequent contacts with infective intestinal parasite stages that may be dispersed in the environment. It could also be due to anthropogenic changes in the community due to logging and fragmentation of the forest which results in the baboons having more routine contact with humans in their search for food coupled with the occasional raiding of homes of the inhabitants. According to Hahn et al. [16], baboons are susceptible to most human pathogenic organisms such as parasites and diarrhoea-causing bacteria; thus, the behaviour of the baboons coupled with anthropogenic changes provide opportunities for an exchange of parasites among the baboons and with humans even though cross-species infection is not certain and needs some molecular verification.

*Entamoeba histolytica* is a protozoan parasite responsible for disease amoebiasis [17]. It has been estimated that about 50 million people are infected worldwide, with approximately 40,000–100,000 deaths annually [18]. Infections caused by *Entamoeba histolytica* is a public health concern in developing countries due to poor personal hygiene and lack of proper sanitation [19]. Little attention has been given to the risk of zoonotic transmission posing a threat to public health [20]. In environments where animals and humans interact, it is essential to understand the risk of zoonotic disease transmission [21]. Changes in an anthropogenic habitat will cause humans and nonhuman primates to have frequent contact leading to an increased risk of disease transmission [22]. A recent study conducted in the Greater Gombe Ecosystem, Tanzania, has confirmed the risk of zoonotic transmission of *Entamoeba histolytica* [23]. Identifying *Entamoeba histolytica*/*dispar* in the baboon faecal samples from Shai Hills Reserve indicates a need to access the potential zoonotic transmission to individuals in the surrounding communities.

The hookworm prevalence rate of 38.39% conforms to other studies [24, 25] which reported a higher occurrence of helminth parasites in the gut of primates. The differences in the prevalence of these parasites could be attributed to differences in transmission dynamics as well as the environmental, parasite, and host factors that contribute to development, survival, and dispersal of the infective stages of the parasite in the environment. Multiparasitism was observed in the baboons, dominated by coinfections; however, no one sample had all the eight parasites. Multiparasitism appeared to be common and is consistent with the results of a study by Mutani et al. [26] which reported 92.5% polyparasitism in all monkeys examined.

The high prevalence of hookworm in the study population is a source of concern, since heavy infections with this parasite have been associated with mucosal inflammation, ulceration, iron deficiency anaemia, protein malnutrition, dysentery, weight loss, and death in primates [27]. Hookworm prevalence compared to the other helminth parasites in this study could be attributed to the fact that hookworm transmission generally occurs in the fields as opposed to near houses as in the case of *A. lumbricoides* and *T. trichiura* [28]. Hookworm is generally transmitted actively by its infective larvae which penetrate through the skin; however, passive transmission can occur through contaminated food or soil that is licked or swallowed by the host. Hookworm theoretically has a two-way transmission of the infective stage, which could account for the high prevalence recorded in this study once the infective stages are dispersed in soil. The dispersal is facilitated as some of the indigenes tend to defecate in the “open bush”; thus, the parasite stages in the faeces are dispersed in the environment in which the baboons are found resting, playing, and uprooting plants for feeding.

The low prevalence of protozoan parasites may be attributed to the dry harsh conditions in the park during the dry period (the season within which this survey was conducted) that favours nematode transmission and development more than protozoans [29]. It is probable that these protozoans are not able to withstand the harsh conditions of the environment and may die off rapidly if they are not able to encyst. It may also be low as a result of the preservation and identification methods used which might have killed the cysts and trophozoites [30].

Intestinal parasites in baboons could be of a permanent occurrence with significant morbidity. However, some of the plants consumed by the baboons as food may be medicinal with active ingredients against some of the parasites [31]; thus, although the baboons may be harbouring these parasites, they may not suffer serious complications.

The adult baboons use their fingers to uproot plants and give it to the juveniles in addition to what they would eat, thus making them more prone to these soil-transmitted infections. Furthermore, the susceptibility to infections is one of the costs associated with dominance [32]. The prevalence of *A. lumbricoides* in adults was comparatively higher than that of the juveniles and subadults. The juvenile had the highest prevalence of hookworm infection than adults and subadults. This could be due to the fact the juveniles move around when the troop is resting, picking up anything on the ground be it food or not. They can even move to the territories of other animals and run back when frightened. The high prevalence may also be attributed to their low level of resistance to parasite development compared to adults. *D. latum* had the lowest prevalence probably because the parasite is waterborne and is transmitted by consuming contaminated fish. Only baboons located close to the dam were observed to have this infection, hence its low prevalence. They drink from the dam and probably eat some of the fishes in the water since this parasite is transmitted from uncooked fish. Detecting *D. latum* infections in the baboons could mean a potential transmission to the human population of the area. It also suggests that individuals who consume fish from the dam are at a greater risk of infections, although this needs to be confirmed by detecting infections in the fish population of the dam.

The strength of this study is that the baboons were tailed and samples collected to ensure each stool sample came from a unique baboon. This prevented sampling twice from the same animal. Furthermore, this study provides information on the distribution of zoonotic gastrointestinal parasites in baboons. To the best of our knowledge, this is the first report of zoonotic parasite distribution at the Shai Hills Reserve. The method employed in this study can be used for the morphological identification of intestinal parasites to at least the genus level. However, it is recommended that future studies make use of molecular methods due to their specificity and sensitivity. It will also be important to educate inhabitants and tourists' on standard hygiene practices within the park to prevent infections.

## 5. Conclusions

This study reveals that baboons at the Shai Hills are infected by gastrointestinal parasites that also infect humans. The high prevalence (92%) of intestinal parasites in these nonhuman primates may serve as reservoirs for some gastrointestinal parasites that infect man, and this calls for management practices to prevent and control these infections.

## Figures and Tables

**Table 1:** Prevalence of various parasites in the different groups.

Type of parasite	Prevalence (%) in various groups		
	Adult	Subadult	Juvenile
*A. lumbricoides*	35.4	10.4	8.3
*S. stercoralis*	0	8.3	2.1
Hookworm	20.9	12.5	47.9
*T. trichiura*	20.1	6.3	20.8
*E. coli*	10.4	4.2	0
*E. histolytica*/*dispar*	10.4	4.2	1
*D. latum*	6	2.1	0
*I. buetschlii*	2.1	2.1	0

## Data Availability

Included in this article are all the data used in supporting the findings.
